# Analgesic Effect of Valerian Root and Turnip Extracts 

**DOI:** 10.29252/wjps.7.3.345

**Published:** 2018-09

**Authors:** Afshin Zare, Zabihollah Khaksar, Zahra Sobhani, Masood Amini

**Affiliations:** 1 Laparoscopy Research Center, Department of Surgery, School of Medicine, Shiraz University of Medical Sciences, Shiraz, Iran;; 2 Department of Basic Sciences, School of Veterinary Medicine, Shiraz University, Shiraz, Iran

**Keywords:** Analgesia, Valerian root, Turnip, Formalin test, Pain, Rat

## Abstract

**BACKGROUND:**

Medicinal plants are considered as one of the important sources of chemical substances with therapeutic effects. This study aimed to compare the analgesic effects of alcoholic extract of valerian root and turnip in rats.

**METHODS:**

Fifty female Wistar rats weighing 190 g were divided into 5 equal groups of control (subcutaneous injection of 2.5% formalin in the right foot), sham (subcutaneous injection of 2.5% formalin+distilled water), experimental 1 (subcutaneous injection of 2.5% formalin+200 mg/kg turnip extract), experimental 2 (subcutaneous injection of 2.5% formalin 2+200 mg/kg valerian root extract) and experimental 3 (subcutaneous injection of 2.5% formalin+200 mg/kg turnip extract+200 mg/kg valerian root extract). The time duration of 0-5 and 16-60 minutes after injection of formalin were respectively considered as acute and chronic phases. Injection of distilled water and the extracts was conducted 30 minutes before assessing the analgesic effects.

**RESULTS:**

A significant decrease in pain score in the acute phase was observed in the group received valerian root extract compared to the control group. Also, a significant reduction in pain score was noted in the acute and chronic phases of the group receiving simultaneous administration of valerian root and turnip extracts when compared to the control group.

**CONCLUSION:**

Simultaneous use of valerian root and turnip extracts is recommended for analgesic effects in both acute and chronic phases of the pain.

## INTRODUCTION

Since control of pain and its complications are important issue in surgical interventions, several and various researches were performed worldwide to find the best way to deal with pain reduction. The pathways involved in induction of pain are largely known, while a large number of medications have been used to control these pathways^1^^-^^4^ and among them, opioid analgesics have been widely used.^5^ Of course it should be borne in mind that these medicines induce tolerance and dependences too, and can cause incidence of unwanted effects; so necessary caution should be undertaken when they are taken by a patient.^6^^,^^7^

These medicines control pain by affecting the central parts of the body. Unfortunately, opioids have their own side effects too and cause gastrointestinal complications such as gastrointestinal bleeding denoting to their hazards when are used.^8^^-^^10^ That is why finding new medicines to alleviate pain and to have fewer side effects than existing medications is very important and essential. Medicinal herbs are the most important and under investigation resources for pain relief that have been traditionally recognized as the important sources of treatment by human being. Nowadays due to the ease of access to these medicines, there is high focus on their use and research.^11^^,^^12^

A medicinal plant which has been received much more attention in traditional medicine is turnip. This valuable plant has long had a special place in traditional medicine and in treatment of diseases. Turnip with scientific name of *brassica rapa* is from *Braaicacaceae* family.^13^ Effective ingredients in this plant are riboflavin, phenols, flavonoids, ascorbic acid, vitamins and potassium.^13^ It has therapeutic properties in respiratory, renal, cutaneous, digestive and joint disorders too.^13^ It has been stated in other studies that turnip extract has anti-allergic, anti-inflammatory, anti-microbial and anti-cancer properties.^14^

Another herb which has also many therapeutic effects in traditional medicine is valerian root. This plant with the scientific name of *Valeriana officinalis* is an herbaceous plant. Among different parts of the plant, the root and rhizomes are used.^15^^,^^16^ Valerian root with valerenic acids and flavonoids is used as a sedative and antispasmodic plant and has been widely used in France, Sweden and Germany for its anti-spasticity and analgesic properties.^17^^,^^18^

The effects of flavonoids available in the extract of valerian and turnip on the synthesis of prostaglandins were clearly indicated. In the process of prostaglandin synthesis from arachidonic acid which occurs in response to inflammatory stimuli, flavonoids were shown to inhibit cyclooxygenase (COX) enzyme (AA) preventing sensitization of pain receptors and reduce the pain sensation associated with the response.^19^^,^^20^ So this study aimed to compare the analgesic effects of alcoholic extracts of valerian root and turnip in female Wistar rats using formalin test. 

## MATERIALS AND METHODS

Fifty adult female Wistar rats weighing approximately 1905 g prepared from Laboratory Animal Center of Shiraz University of Medical Sciences were divided to 5 equal groups. The control group just underwent formalin test without any other intervention and the sham group received 1 ml of distilled water intraperitoneally, 30 minutes before intraperitoneal injection of formalin. The 1^st^ experimental group received 200 mg/kg of alcoholic extract of turnip root intraperitoneally, 30 minutes before injection of formalin. The 2^nd^ experimental group received 200 mg/kg of alcoholic extract of valerian root was intraperitoneally, 30 minutes before injection of formalin. The 3^rd^ experimental group received 200 mg/kg of alcoholic extract of valerian root together with 200 mg/kg of alcoholic extract of turnip root intraperitoneally. 30 minutes before injection of formalin. All animals were kept under identical conditions at 22 ˚C and a period of 12 hours of light and 12 hours of darkness and fed with adequate water and food as described before.^21^

To prepare turnip extract, 100 g of turnip roots were thoroughly washed and cut, and then ethanol was used three times in the extraction process. The resulting was filtered and completely dried with a vacuum rotary device. The extract was kept in refrigerator before use as reported before.^22^ To provide valerian root extract, the roots were changed into powder using electrical mill based on Soxhlet method adding 200 ml of related solvent containing water and ethanol to every 10 g of valerian root powder. The solution was then transferred to Soxhlet device and using a Rota vapor device. The solvent was finally removed from the extract at the end of the process. 

All tests were performed between 7:30 AM to 16 PM. Half an hour prior to each experiment, the rats were transferred from cages to glass chambers for formalin test to adopt with their environment. Each animal was tested only once and euthanized by diethyl ether at the end of the experiment. For formalin test, 0.05 ml of 2.5% formalin was injected subcutaneously into the right hind paw of the rats as explained before.^23^

The animals were immediately placed in the test chamber and pain behavior was assessed using a mirror embedded at 45 degree angle to the horizon surface in the lower section of the chamber. Every 15 seconds, the behavioral response was recorded. Animal pain intensity was defined as 4 degrees based on the conventional category as Zero: When the animal had complete balance in walking and the weight was distributed equally on each leg; One: When the animal did not tolerate the body weight on the injected paw and/or when a problem existed with walking; Two: When the animal raised the painful toe and had no contact with the floor of the chamber; and Three: When the animal licked and/or severely shacked the painful paw.

Recording of behavioral responses began immediately after injection of formalin and was continued for 60 minutes. Pain score was calculated as 12 blocks of 5 minutes during 60 minutes period of the experiment according to the formula. Where, T_0_, T_1_, T_2_ and T_3_ are the number of 15 seconds in which the animal shows, respectively 0, 1, 2 and 3 behaviors in a period of 5 minutes. The time duration of 0-5 and 16-60 minutes were considered as acute and chronic phases for all groups, respectively. SPSS software (version 18, Chicago, IL, USA) using ANOVA was used for statistical analysis. The statistical difference between groups was considered as P<0.05. 

## RESULTS

According to the results, a reduction in pain score for both acute and chronic phases was observed in the group receiving turnip extract when compared to the control group. This difference was not statistically significant. A significant reduction in the pain score in acute phase was noted in the group receiving valerian extract when compared to the control group. The simultaneous use of both valerian and turnip extracts could significantly reduce the pain score in both acute and chronic phases when compared to other groups (Table 1). 

**Table 1:** The comparison of the pain score in the groups receiving various amounts of alcoholic extracts (valerian root and turnip) with the control group in both acute and chronic phases of pain

**Group**	**Acute pain**	**Chronic pain**
2.5% Formalin	2.24±0.097	1.72±0.114
2.5% Formalin+Distilled water	2.29±0.165	1.70±0.039
2.5% Formalin+Turnip extract (200 mg/kg)	1.9±0.083	1.47±0.112
2.5% Formalin+Valerian root extract (200 mg/kg)	1.66±0.133*	1.46±0.101
2.5% Formalin+Turnip extract (200 mg/kg)+Valerian root extract (200 mg/kg)	1.45±0.132*	1.12±0.054*

Available mean in each square which has an asterisk (*) indicates significant difference with the control group (*p*<0.05).

## DISCUSSION

Our results showed a reduction in pain score in the group receiving turnip extract and the group receiving valerian root extract in comparison to the control group. The findings show the analgesic effects of the two extracts. When both extracts were simultaneously used, the analgesic effect was more prominent. It was demonstrated that nitric oxide (NO) plays a crucial role in mediating of many functions in nervous system including the memory formation, sexual feeling, aggressive and nutritional behaviors.^24^ Nitric oxide as a free radical messenger has high ability of emission by a number of cells. It increases membrane permeability and its role in pain has also been taken into consideration as the level of nitric oxide increases in the injured area following the damage in nervous system.^25^^,^^26^

Nitric oxide is considered as an active messenger in induction of pain. It is synthesized by nitric oxide synthase from L-arginine, while three types of nitric oxide synthase have been identified till now.^27^ The mechanism of action of nitric oxide in pain process based on activation of neuronal nitric oxide due to tissue damage or inflammation.^28^ It has been suggested that turnip extract has active biological compounds such as (i) flavonoids including isorhamnetin, kaempferol and quercetin glycosides; (ii) phenylpropanoid derivatives; (iii) indole alkaloids; (iv) sterol glycosides; and (v) ascorbic acid (139 mg), vitamin A, niacin and riboflavin.^13^^,^^29^

As valerian root extract has flavonoids and phenolic compounds,^17^^,^^18^ flavonoids are responsible for inhibition of nitric oxide synthesis as well as synthesis of protein kinase C and prostaglandin E_2_.^30^ The flavonoids were shown to prevent production of NO after injection of formalin leading to an analgesic activity.^14^^,^^31^ Since NO is a hyperalgesia mediator,^23^ if declines, it would lead to an analgesic property. Similar medicinal compounds and plants with flavonoids were shown to decline inflammatory responses of formalin test by influencing on synthesis of prostaglandins (E2) and their decrease.^32^^,^^33^

The reduction of pain, inflammation and pain in acute phase would decline the plasticity; and practical changes in dorsal branch of the spinal cord and would reduce the release of transmitters such as P substance and excitatory amino acids from the end of nerve fibers similar to our findings.^34^^,^^35^ In another study conducted on plants with flavonoids, it was shown that available flavonoids in the plants inhibit pain.^36^ As inflammation is associated with production of pain in the second phase of formalin test, so a part of analgesic effects of flavonoids is related to the anti-inflammatory properties of them,^37^

In the current study, valerian root and turnip extracts lead to a reduction in pain score using formalin test, that can be due to the presence of flavonoid compounds and the influence on prostaglandins as well as their anti-inflammatory properties. Similar study on plants with similar ingredients has shown that plants with flavonoids can inhibit cyclooxygenase (COX) in human monocytes and reduce the synthesis of prostaglandin E or thromboxane B2.^38^ It was demonstrated that voltage-gate calcium channels play an important role in control of cellular function in various tissues such as heart, blood vessels and nervous system. The evidences indicate that pharmacological blocking of calcium channels may have analgesic effects and be useful in treatment of visceral and somatic pains due to a decline in calcium crossing.^39^

Calcium interferes with release of neurotransmitters and other materials which promote pain and inflammation. The activation of calcium channels is dependent on depolarization of membrane and neurotransmitters, and various materials are released by entrance of calcium causing the pain behavior.^39^ Calcium channel blockers are recently taken into consideration as analgesic factors and it has suggested that some blockers of calcium channels have analgesic effect in pre-clinical and clinical models of pain.^40^^,^^41^ Previous studies have suggested that flavonoids reduce intracellular concentration of calcium by inhibiting the activity of N-methyl-D-aspartate receptors, activities of nitric oxide synthase enzyme and calcium-dependent phospholipase A2 that are reduced. So flavonoids exert their analgesic effect by reduction in concentration of NO and prostaglandins.^42^ The results of our study also denote to a decline in the pain score in the groups received the extracts of valerian root and turnip when compared to the control group. In the case of simultaneous use of the extracts, this effect was more prominent. 

Based on the results of our study, the hydroalcoholic extracts of valerian root and turnip in the experimental groups lead to a significant reduction in pain score when compared to the control group using the formalin test. The extracts of valerian root and turnip had anti-inflammatory and analgesic properties that can be due to presence of flavonoid compounds in these plants, the influence of prostaglandins as well as cyclooxygenase enzyme and the decline in intracellular calcium. 
